# Improved angle accuracy of tibial plateau osteotomy for total knee arthroplasty using tibial mechanical axis skin-mapping

**DOI:** 10.3389/fsurg.2022.961667

**Published:** 2023-01-06

**Authors:** Peiheng He, Shuai Huang, Yong Liu, Xing Li, Dongliang Xu

**Affiliations:** Department of Joint Surgery, Guangdong Provincial Key Laboratory of Orthopedics and Traumatology, The First Affiliated Hospital of Sun Yat-sen University, Guangzhou, China

**Keywords:** middle tibia crest line, tibia mechanical axis, skin mark, tibia plateau osteotomy, total knee arthroplasty

## Abstract

**Background:**

The tibial crest is often used as an anatomic landmark for tibial plateau osteotomy (TPO) in total knee arthroplasty (TKA), but it is not very accurate. This study aimed to investigate errors in using the tibial crest as a marker and present a simple approach to improve the angle accuracy of TPO by mapping the tibial mechanical axis (TMA), determined preoperatively, according to the tibial crest on the skin overlying the tibia.

**Methods:**

We evaluated 50 healthy young volunteers and 100 pre-TKA osteoarthritic knees. The middle tibial crest lines (MTCLs) were marked on the shank tibial skin and covered with Kirschner wires. All participants underwent two sets of anteroposterior (AP) standing radiographs of the lower extremity, with the feet in neutral and external rotation positions. The MTCL–TMA angles were measured and compared. The TMA was mapped onto the tibial skin according to the MTCL–TMA angle prior to TKA and used for TPO. Postoperative outcomes were determined by the angle between the vertical tibial component axis (TCA) and the TMA.

**Results:**

The MTCL had no evident relationship with the TMA. A few MTCLs were parallel to the TMA. External rotation of the foot significantly changed the MTCL–TMA relationship. The angle accuracy of the TPO as guided by TMA skin-mapping was 0.83 ± 0.76°. No postoperative errors exceeded 3°.

**Conclusion:**

The MTCL was not equivalent to the TMA. The TPO error can be reduced by preoperatively marking the TMA on the tibial skin according to the MTCL.

## Introduction

In total knee arthroplasty (TKA), proper postoperative limb alignment is critical for satisfactory clinical outcomes and implant longevity (1, 2). Neutral mechanical alignment (MA) in the coronal plane has been the primary objective of knee replacement for decades (3). Approximately 20% of patients remain dissatisfied after TKA, some because of the varus/valgus malalignment of the tibial and/or femoral components that results from inaccurate osteotomy (4). Recently, this standard has been challenged. Some studies have reported that patients with preoperative varus knees had better postoperative functional scores when a slight varus alignment was retained (5, 6). However, the short-term alignment after TKA may change with time. One study reported that a varus position of the femoral and tibial prostheses might induce varus alignment progression 10 years after TKA (7). Moreover, conventional prostheses with patient-specific alignment can lead to bone–implant mismatch or affect patellofemoral kinematics (8). Currently, most surgeons insist on the importance of obtaining a neutral MA. Whether a slight varus position or patient-specific alignment is preferable should be further verified through multicenter randomized controlled trials.

Postoperative MA is attained via femoral and tibial plateau osteotomy (TPO) during TKA. For TKA, either measured resection or gap balancing is implemented. Both techniques are based on the accuracy of the TPO in the coronal plane. Recent research has found TPO accuracy to be an independent predictor of postoperative alignment (9). TPO can be performed using an intramedullary guide or extramedullary guide (EMG); the latter are more commonly used (10). To date, many anatomical landmarks have been used to aid EMG (11). However, only 60%–80% of surgeries using EMGs have obtained TPO accuracy within 3° in the coronal plane (12, 13).

The tibial crest is covered only by skin. Therefore, it is a convenient anatomical marker for EMG. Fukagawa et al. reported that the tibial crest was almost parallel to the tibia mechanical axis (TMA) (14). However, Cinotti et al. found that the tibial crests of Caucasian patients exhibited marked variability from the TMA; thus, they opposed the use of the tibial crest as a primary anatomical landmark for tibial coronal alignment in these patients (15). The accuracy of EMG based on the tibial crest thus remains controversial.

All preoperative and postoperative MA assessments are performed using anteroposterior (AP) standing radiographs of the lower extremities. Patients are instructed to keep the knee extended with the patella facing directly anterior, with the ankle in a neutral position to control rotation. However, this is challenging in practice because of patellar subluxation or tilt, and because of the influence of foot rotation. Further, it is difficult to achieve comparability and repeatability between two patients or even in the same individual at different time points.

Therefore, this study aimed to investigate the relationship between TMA and middle tibial crest lines (MTCLs) according to images taken with the foot in neutral and external rotation, and to present a simple approach to promote accurate TPO during TKA by marking the TMA on the skin overlying the tibia according to the MTCL.

## Materials and methods

### Participants

A total of 50 healthy volunteers and 100 patients with knee osteoarthritis were recruited for this study at our hospital between January 2015 and December 2019. The volunteers were 20–25 years old and in good health. The mean age of the patients was 70.8 ± 9.37 years, and the patient cohort comprised 15 male and 85 female knees. The included patients had primary osteoarthritic knees in which the varus or valgus deformity was not more than 15°, according to the hip–knee–ankle (HKA) angle. Patients undergoing revision TKA, as well as those with infection or a previous history of knee surgery, were excluded from the study.

The study protocol was reviewed and approved by the Institutional Review Board and Ethics Committee of the First Affiliated Hospital of Sun Yat-sen University [code number: (2011) 58]. Written informed consent was obtained from all participants prior to enrollment.

### Radiographic measurements

**Figure 1:**
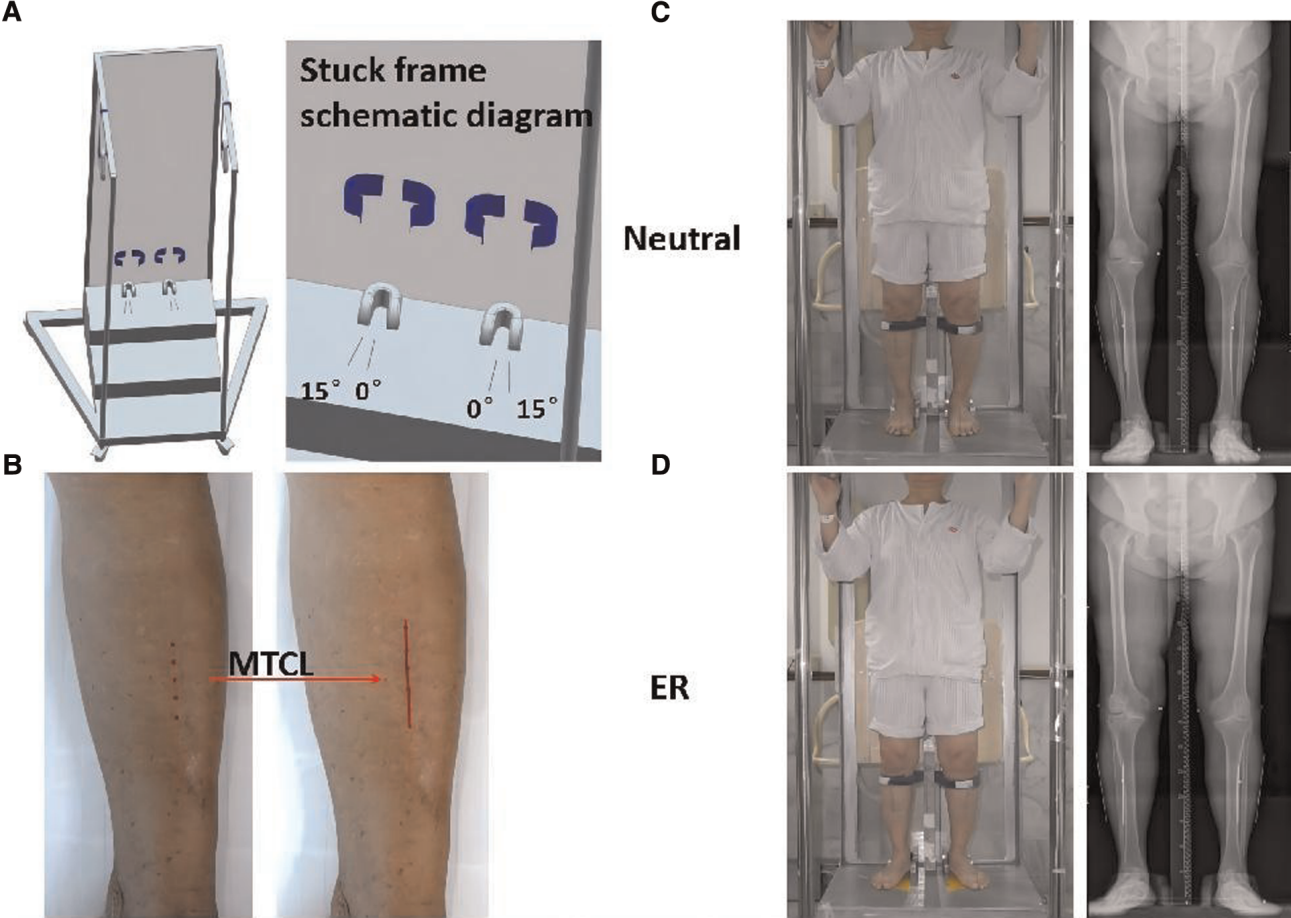
Participants underwent anteroposterior (AP) standing radiographs of the lower extremities. (**A**) Secure frame schematic diagram. The feet are aligned on the platform with the center between the toes and heels of feet I and II, and the superior tibial crest is secured. (**B**) The middle tibial crest line (MTCL) is marked on the skin (red line). (**C,D**) A secure frame is used to perform radiographs: feet positioned straight ahead (neutral) and at 15° of external rotation (ER).

The MTCLs were drawn on the skin connecting the two points on the tibial crest (10 cm and 20 cm distal to the knee joint line). Kirschner wires were then attached to the skin along the MTCL. AP standing radiographs of the lower extremities were obtained with the participants’ feet fixed in a neutral position (the heels and first toes of the right and left sides parallel, considered 0°) and again with the feet in 15° of external rotation (ER) within a secure frame (Figure 1). The x-ray beam was positioned perpendicular to the detector and was directed at the center of the knee from a distance of 2 m. The voltage and current were set at 85 kV and 200 mA, respectively. All measurements were obtained using Picture Archiving and Communication Systems workstation software (PACS 2.0.4.15, Annet Information System).

**Figure 2:**
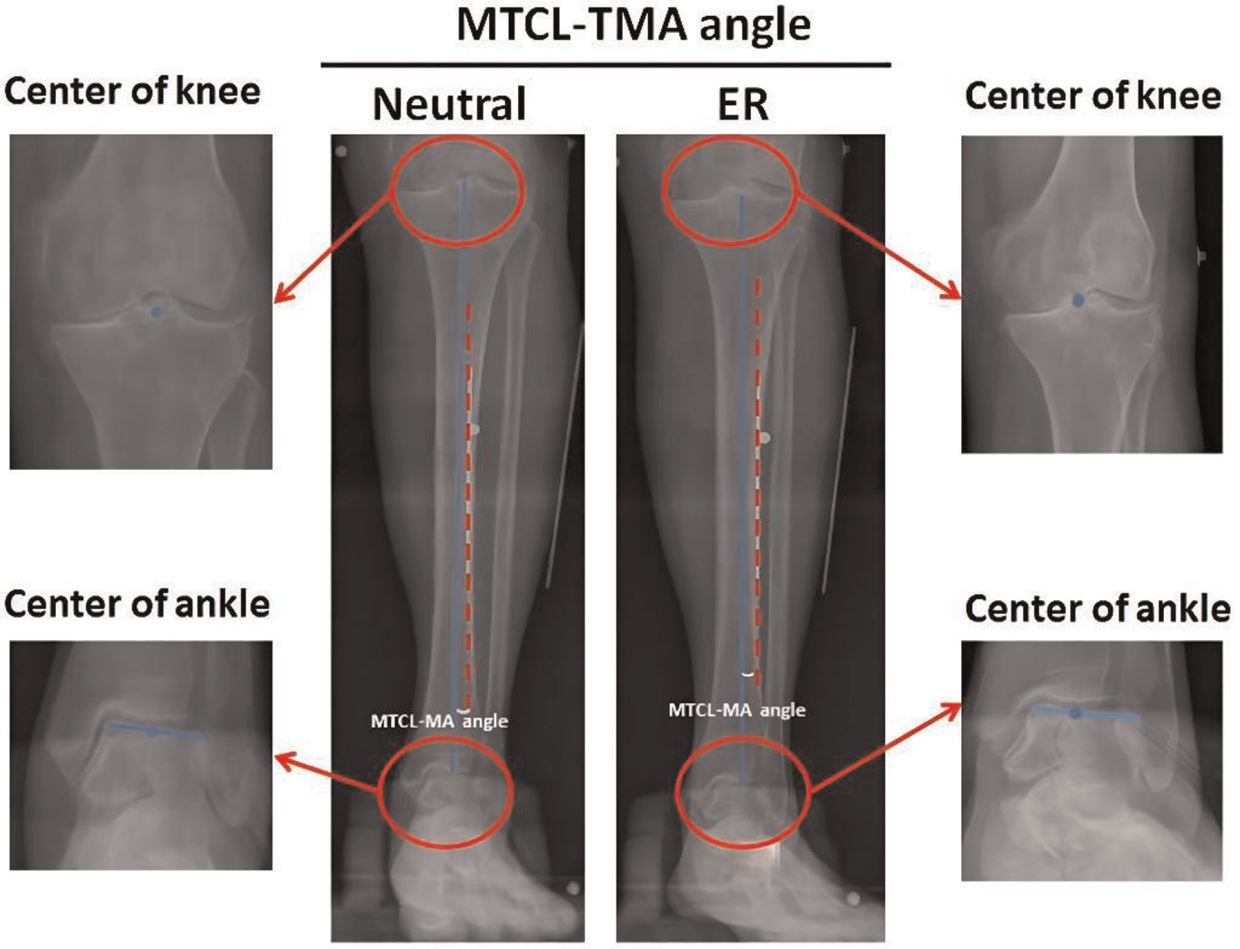
Measurement of the MTCL–TMA angles. The tibial mechanical axis (TMA) (blue line) is defined by connecting the points between the centers (blue dots) of the knee and ankle. The MTCL is defined as the red dotted line. The MTCL–TMA angle was automatically calculated by PACS software.

The TMA was defined as the straight line from the center of the knee to the center of the ankle (16). Digitalized radiographs were collected to analyze the relationship between the MTCL and TMA. The MTCL–TMA angles were measured in the coronal plane at two positions (Figure 2). This angle was defined as varus or valgus when the MTCL was pointed distally toward the lateral or medial malleolus, respectively.

### TMA skin-mapping before TKA

**Figure 3:**
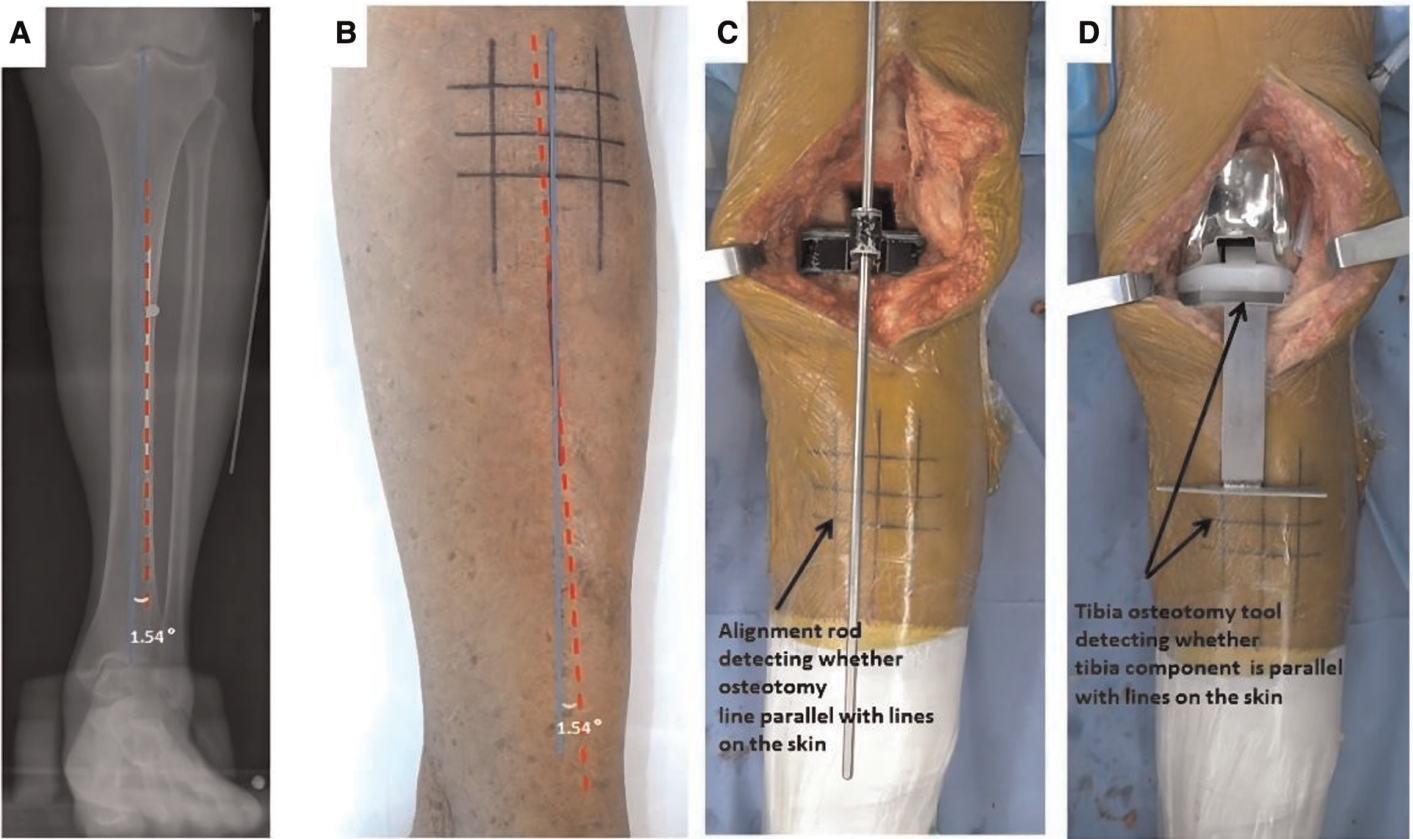
The TMA mapped on the skin before TKA surgery. (**A**) The MTCL–TMA angle is measured by PACS software. (**B**) The TMA lines are marked on the skin, modulated by the MTCL–TMA angle. The parallel and vertical TMA lines are marked for TKA. (**C**) After tibial osteotomy, an alignment rod is used to detect whether the alignment matches the skin markings. (**D**) The position of the tibial component is double-checked using a specialized tool.

**Figure 4:**
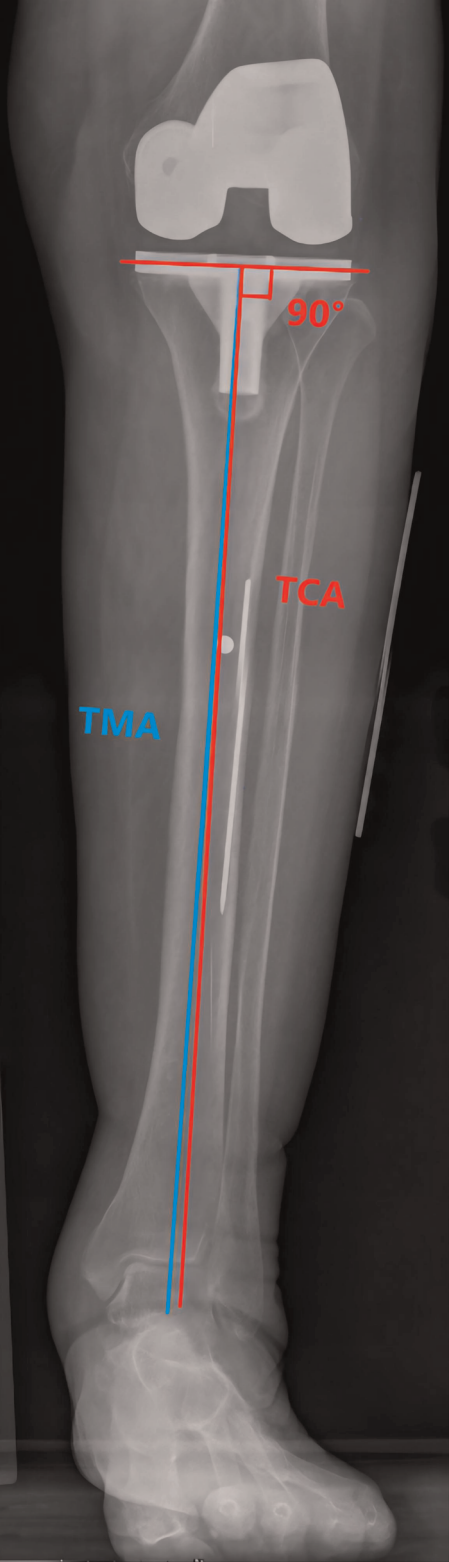
The TMA–TCA angle is used to detect the accuracy of the tibial osteotomy. The TCA (red line) is defined as the vertical line through the tibial component tray.

Preoperative MTCL–TMA angles were measured using PACS. The TMA was then marked on the front side of the skin overlying the tibia according to the marked MTCL. For TKA, the TMA was marked with three parallel and three vertical lines (Figure 3). The conventional operative approach was used. TPO was performed using an EMG jig. The EMG jig rotation was parallel to the AP axis of Akagi et al. (17), and the jig was then oriented parallel to the TMA reference line on the front side of the tibial skin in the coronal plane, reproducing a 3° posterior inclination in the sagittal plane. TPO was subsequently performed, and the cut plane was determined based on the alignment rod, which was parallel to the TMA reference line (Figure 3). A cemented PFC Sigma Knee System (DePuy Synthes, Johnson & Johnson, Warsaw, IN, USA) was used. After cementing the total knee components, the tibial component was double-checked using a specialized tool to determine whether it matched the three horizontal lines (Figure 3). The TPO accuracy was presented as the angle between the vertical tibial component axis (TCA) and the TMA on postoperative projection (Figure 4).

### Data analysis

Intra- and interobserver measurement reliabilities were assessed using the intraclass correlation coefficients (ICCs). To test intra- or interobserver reliability, all measurements were repeated three times by two observers with a 2-week interval between measurements, and the data were expressed as mean ± SD (SEM). We used Student's *t*-test to compare the angle measurement differences for each dataset. Statistical significance was defined as *p* < 0.05. All statistical analyses were performed using the Statistical Package for the Social Sciences (SPSS), version 17.0, for Windows (SPSS, Chicago, IL).

## Results

### Evaluation of reproducibility

Measurement reliability was excellent for the angles between TMA and other axes, including the MTCL, TCA, and femoral mechanical axis (FMA). Intra- and interclass correlation coefficients were 94%–99%, respectively (Table 1).

**Table 1:** Intraclass correlation coefficient of interobserver and intra-observer error of all parameters.

	Intraobserver	Interobserver
	ICC	ICC
TMA–MTCL angle	0.979	0.952
TMA–TCA angle	0.980	0.959
HKA angle	0.990	0.968

TMA, tibia mechanical axis; MTCL, middle tibia crest line; TCA, tibial component axis; HKA, hip-knee-ankle.

### Respective locations of TMA and MTCL

**Figure 5:**
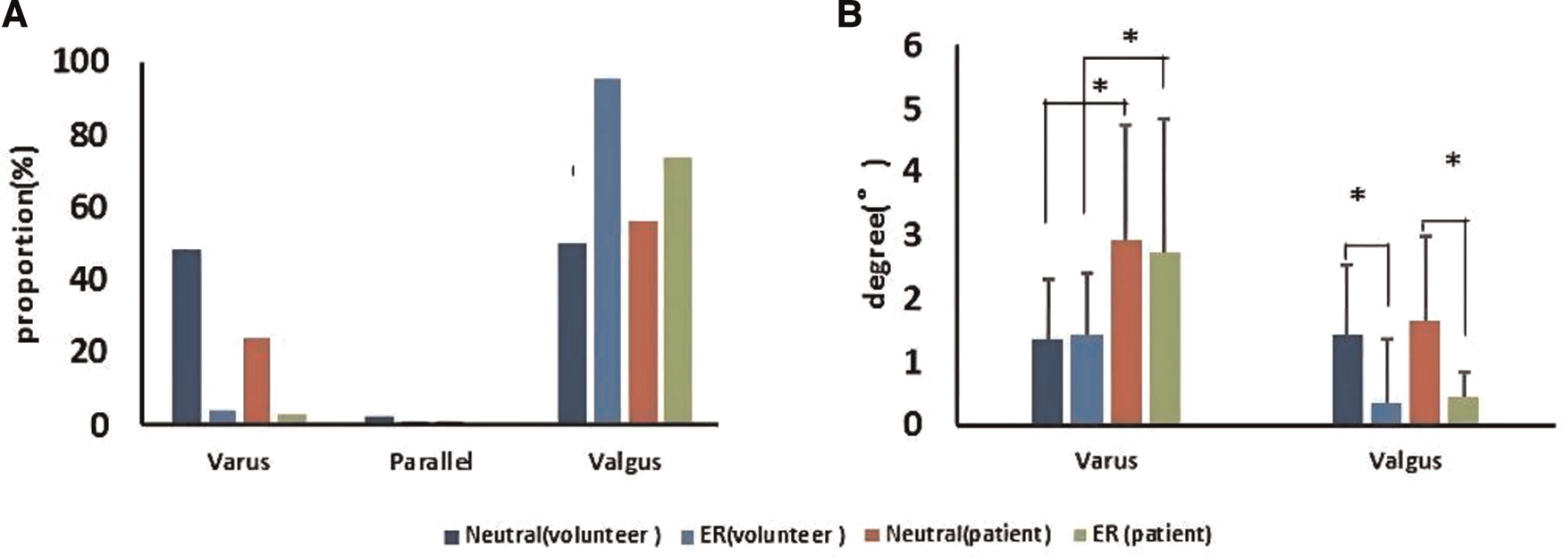
The MTCL–TMA angle in volunteers and patients, in neutral and ER positions. (**A**) Proportions of the MTCL–TMA angle. (**B**) Ranges of the MTCL–TMA angle measurements (*p < 0.05).

The MTCL had no evident relationship with the TMA. A few MTCLs were parallel to the TMA (Figure 5). The proportions of MTCL–TMA angles exceeding 3° in the neutral or ER position were 16.0% and 18.0%, respectively, among volunteers and 23.0% and 25.0%, respectively, among patients. Therefore, MTCL was less reliable during surgery. The ER foot position significantly changed the relationship between the MTCL and TMA. The MTCL–TMA angles appeared more varus among the healthy participants, but more valgus among the patients (Figure 5). However, among patients, the varus degree of the MTCL–TMA angles was greater than that of the valgus degree (Figure 5), which might partially explain why an abnormal varus position of the tibial component always occurred.

### Accuracy of TMA skin marking for TPO

**Figure 6:**
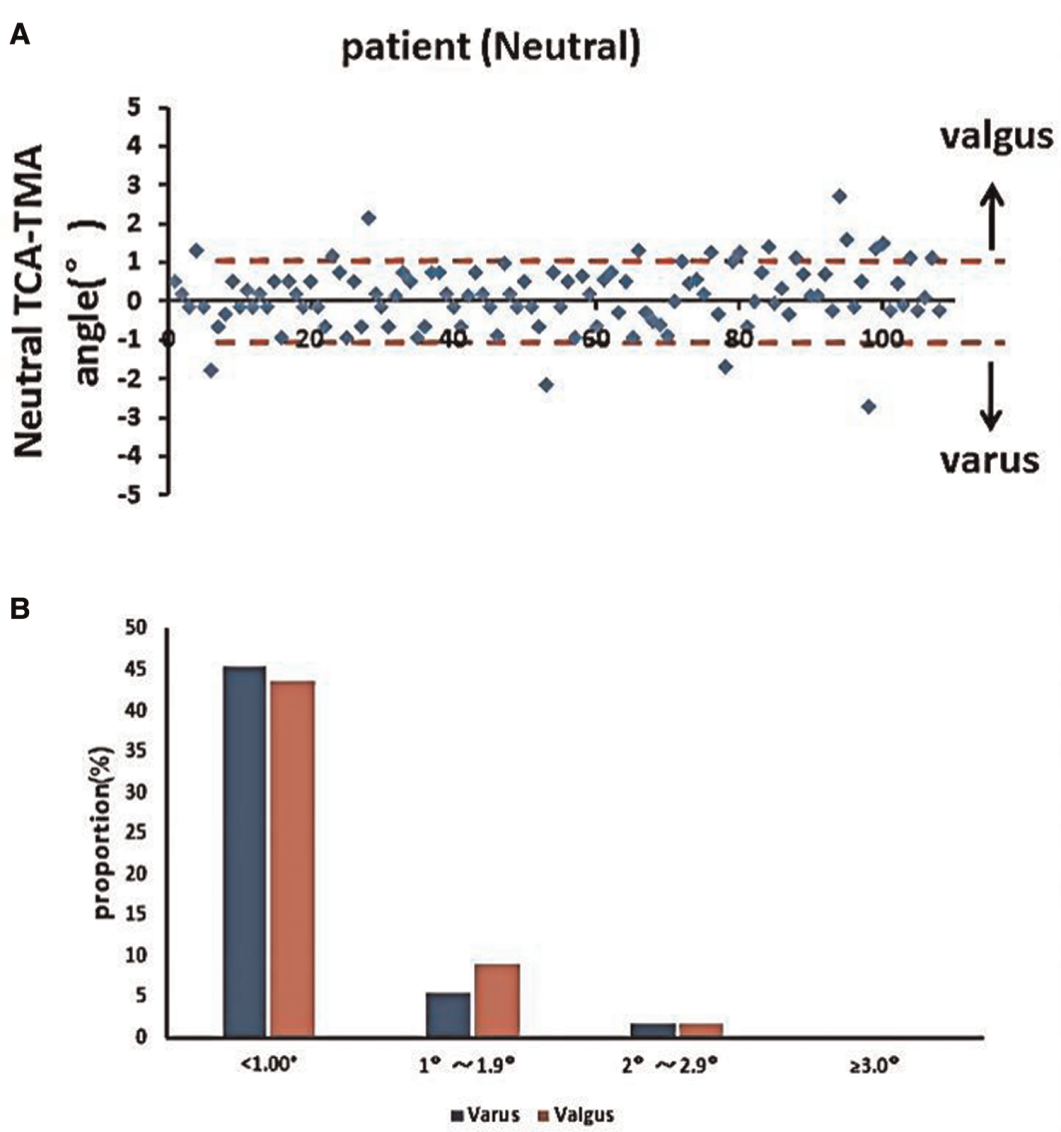
Accuracy of TMA mapping on the tibial skin for TPO. (**A**) Distribution of TMA–TCA angles in the neutral position. (**B**) Proportions of TMA–TCA angles after TKA (*p < 0.05).

The accuracy of the TPO guided by the TMA marked on the tibial skin was measured as the postoperative angle of TCA and TMA, which was 0.83° ± 0.76°. No case exceeded a 3° error (Figure 6). The coronal tibial component showed improved accuracy.

## Discussion

In this study, the MTCL had no evident relationship with the TMA. A few MTCLs were parallel to the TMA. The ER foot position significantly changed the relationship between MTCL and TMA.

To the best of our knowledge, this was the first study to assess the relationship between MTCL and TMA in patients and healthy young adults via reproducible radiographs using a secure frame to hold specific rotation angles of the feet. Consequently, this study offers a potential method for TMA orientation. This method produced minimal error when marking the TMA on the tibial skin. The TMA was determined preoperatively according to the MTCL.

During TKA, the surgeon must estimate the coronal, sagittal, and axial alignments of the prosthesis. The major argument regarding alignment continues to support a neutral mechanical or kinematic alignment. Some scholars have found that kinematically aligned knees in slight varus had better postoperative functional outcomes, including a higher mean flexion range angle and better pain relief and stability (18, 19). However, published outcomes have used only short-term follow-up. A meta-analysis found that postoperative varus limb alignment was a predictor for increased migration of uncemented prostheses (20). MA has been used in TKA for more than 30 years and continues to be performed worldwide. Coronal MA of the tibial component is primarily achieved using EMG. The goal is to align the EMG with the TMA using the surgeon's subjective judgment. Although the first or second metatarsal bone, tibialis anterior muscle, extensor hallucis longus, and dorsal pedis artery have been used as references for EMG–TMA alignment, all are easily affected by ankle joint position, making it difficult to reproduce TMA. The anterior tibial border is easily palpated and is more practical than the previously mentioned landmarks; however, the reliability of the anterior tibial border remains controversial. Nishikawa et al. reported that the proximal and distal thirds of the anterior tibial borders were highly consistent with TMA in the coronal plane (14). Furthermore, Tadashi et al. identified MTCL as a more useful landmark than those of the conventional method, and patient-specific factors, including tibial bowing and torsion, did not affect its reliability (20). However, we must note wide variations in TMA–MTCL angles among individuals. Cinotti et al.’s investigation showed that the TMA–MTCL angle differed by more than 3° in approximately 35% of cases (15). In this study, the MTCL–TMA angles demonstrated wide variation in both neutral and ER positions (exceeding 3° in 16.0% and 18.0% of volunteers and 23.0% and 25% of patients, respectively).

In contrast to previous research, our study included both patients and young healthy volunteers. Our results show that differences between MTCL and TMA are prevalent regardless of osteoarthritis. Therefore, we cannot recommend MTCL as an independent anatomical reference to guide TMA during TKA, particularly to reduce abnormal positioning of the tibial component. Moreover, in our patient cohort, the degree of varus deviation was greater than the degree of valgus deviation in the measured MTCL–TMA angles. Thus, our results at least partially explain why the varus position of tibial components is more common.

In recent years, orthopedic surgeons have shown great interest in computer-assisted navigation and patient-specific instrumentation (PSI) techniques. These techniques are expected to improve alignment during TKA (21, 22). However, studies have concluded that the new methods were no better than conventional methods (23, 24). Furthermore, some authors pointed out the learning curve imposed by the novel techniques and questioned whether they increased surgical time and cost. Additionally, the anterior border may not be appropriately palpable in some individuals from the previous study, particularly in those with high body mass index.

Based on these drawbacks, we presented a simple approach to improve TPO accuracy by marking the TMA on the tibial skin according to the MTCL. The mean TMA–TCA angle in our study was 0.83° ± 0.76°. Meanwhile, no case had an error exceeding 3°; this was an improvement over previous studies. Patients also underwent AP standing radiography of the lower extremity with a fixed neutral position. This made comparability and repeatability of the tibial component positioning more consistent over time.

Our study had several limitations. First, there were fewer male patients than female patients enrolled, likely because osteoarthritis is more common among women in Asia. Second, the orthopedic surgeons could not reach a consensus on the choice of TMA that provided better clinical outcomes. If the criterion for TMA changes, the method for TPO will also change. The method used in this study was limited to obtaining a desirable TKA alignment according to the surgeons. Third, coronal TMA was applied according to the MTCL, but the sagittal or torsional axis was still applied using conventional methods. In the future, we expect to perform similar preoperative planning in the other two axes to yield more accurate alignment. Fourth, we were not able to assess skin mobility using a specialized quality scale. Even if we draw several perpendicular reference lines on the skin to observe the displacement caused by skin mobility, special care must be taken for patients with slack skin.

## Conclusion

Our study demonstrated that the MTCL was not equivalent to the TMA. TPO inaccuracy can be reduced by preoperatively marking the TMA on the tibial skin based on the MTCL.

## Data Availability

The raw data supporting the conclusions of this article will be made available by the authors, without undue reservation.
